# Supplementary material for: Prevention of Surgery-Induced Dry Eye by Diquafosol Eyedrops after Femtosecond Laser-Assisted Cataract Surgery
Source: J Clin Med. 2022 Sep 28;11(19):5757. doi: 10.3390/jcm11195757 (PMC9572360; doi:10.3390/jcm11195757)
Supplement: Supplementary file 1 [file jcm-11-05757-s001.zip › jcm-1917790-supplementary.pdf]

Table S1. Pre-operative comparison between both eyes.

| Variables | Correlation Coefficients R | (95% CI) | P Value |
| --- | --- | --- | --- |
| UDVA (5m) | 0.28 | (-0.01 – 0.63) | 0.068 |
| CDVA (5m) | 0.26 | (-0.03 – 0.51) | 0.084 |
| UNVA (40cm) | 0.21 | (-0.09 – 0.57) | 0.176 |
| CNVA (40cm) | 0.17 | (0.13 – 0.44) | 0.267 |
| UIVA (70cm) | 0.29 | (0.00 – 0.53) | 0.052 |
| CIVA (70cm) | 0.30 | (0.01 – 0.64) | 0.057 |
| SPK Score | -0.24 | (-0.49 – 0.06) | 0.115 |
| BUT (sec) | -0.21 | (-0.46 – 0.20) | 0.185 |
| Schirmer (mm) | -0.13 | (-0.41 – 0.16) | 0.382 |
| Contrast Sensitivity (3 cpd) | -0.14 | (-0.41 – 0.27) | 0.387 |
| Contrast Sensitivity (6 cpd) | -0.16 | (-0.43 – 0.14) | 0.295 |
| Contrast Sensitivity (12 cpd) | -0.13 | (-0.39 – 0.28) | 0.432 |
| Contrast Sensitivity (18 cpd) | -0.08 | (-0.36 – 0.22) | 0.605 |

R = Pearson’s correlation coefficient; CI = confidence interval; BCVA = Best corrected visual acuity; UCVA = Uncorrected Visual Acuity; SPK = superficial punctate keratitis; BUT = Tear film break-up time
